# Supplementary material for: Effect of utilizing either a self-reported questionnaire or administrative data alone or in combination on the findings of a randomized controlled trial of the long-term effects of antenatal corticosteroids
Source: PLoS One. 2024 Aug 7;19(8):e0308414. doi: 10.1371/journal.pone.0308414 (PMC11305536; doi:10.1371/journal.pone.0308414)
Supplement: S1 Appendix — (DOCX) [file pone.0308414.s001.docx]

# **Appendix 1. Outcome definitions**

In the self-reported questionnaire participants were asked if they had ever been told by a doctor that they had specific diagnoses and what treatment they had for these.

We used the following criteria to define the conditions of interest based on records in administrative datasets:

Diabetes mellitus [42]: Any of:

1. Two haemoglobin A1c (HbA1c) ratios ≥ 50 mmol/mol.
2. Two fasting plasma glucose concentrations ≥ 7.0 mmol/l.
3. A two-hour plasma glucose concentration on a 75 g oral glucose tolerance test ≥ 11.1 mmol/l.
4. One HbA1c ratio ≥ 50 mmol/mol plus one fasting plasma glucose concentration ≥ 7.0 mmol/l.
5. Dispensing records for metformin, insulin or other diabetes medications.
6. Records of attendance at a diabetes clinic or retinal screening for diabetes.
7. Hospital admissions with diagnostic codes for diabetes mellitus.

Pre-diabetes [42]: Any of:

1. One HbA1c ratio 41-49 mmol/mol.
2. One fasting plasma glucose concentration 6.1-6.9 mmol/l.
3. A two-hour plasma glucose concentration on a 75 g oral glucose tolerance test 7.8-11 mmol/l.

Hyperlipidemia [43]: Any of:

1. Total cholesterol concentration > 5 mmol/l.
2. LDL (low-density lipoprotein) cholesterol concentration > 3.4 mmol/l.
3. Triglyceride concentration > 2 mmol/l.
4. Dispensing records for lipid-lowering medications.
5. Hospital admissions with diagnostic codes for hyperlipidemia.

High blood pressure: Any of:

1. Dispensing records for antihypertensive medications.
2. Hospital admissions with diagnostic codes for high blood pressure.

Mental health disorders: Any of:

1. Dispensing records for depression or anxiety medications.
2. Hospital admissions with diagnostic codes for depression or anxiety.

Asthma: Any of:

1. Dispensing records for asthma medications.
2. Hospital admissions with diagnostic codes for asthma.
