# Supplementary material for: Effect of utilizing either a self-reported questionnaire or administrative data alone or in combination on the findings of a randomized controlled trial of the long-term effects of antenatal corticosteroids
Source: PLoS One. 2024 Aug 7;19(8):e0308414. doi: 10.1371/journal.pone.0308414 (PMC11305536; doi:10.1371/journal.pone.0308414)
Supplement: Attachment — Submitted filename: 29Apr2024 Rebuttal Letter.docx [file pone.0308414.s002.docx]

Greg Gamble

Liggins Institute, University of Auckland

85 Park Road, Grafton

Auckland, 1023

New Zealand

+64 27 423 0959

[gd.gamble@auckland.ac.nz / ORCID](mailto:gd.gamble@auckland.ac.nz%20%20/%20ORCID) 0000-0003-0412-3203

Jayeshkumar Patel
Academic Editor
PLOS ONE

29 April 2024

Dear Dr Patel,

**RE: PONE-D-23-40655 Effect of utilizing either a self-reported questionnaire or administrative data alone or in combination on the findings of a randomized controlled trial of the long-term effects of antenatal corticosteroids.**

Thank-you for your invitation to submit a revised version of the above submission. We have carefully addressed each of the points raised by both reviewers and revised the manuscript accordingly. Each of the points raised by the reviewers are listed in the table below alongside our response.As noted by reviewer 1 we believe this manuscript provides insights into outcome ascertainment methods in clinical research and hope that you may consider it for publication.

The authors have no conflicts of interest to declare.

Yours faithfully,

Associate Professor Greg Gamble MSc

Liggins institute

University of Auckland, Auckland, New Zealand

| Comment | Response |
| --- | --- |
| Reviewer 1 | |
| Please separate the data source section and section describing data sources and what variables was used from each one including the self-reported questionnaire. | We added a data sources section subheading, and a table (Table 1) describing all datasets. |
| How missing data were handled or the potential implications of missing data would strengthen the analysis. | For all administrative datasets, the absence of any confirmatory evidence for a specific condition was assumed to represent no evidence for that condition. This is stated in the second paragraph of the “Data sources” section of the methods. |
| Please add a sentence about the descriptive statistics. | This has been added to the first sentence of the “Statistical analysis” section. |
| Clarification is needed regarding the statistical test used to generate the p-value in table one. | Added to the legend of table 2 (previously table 1). |
| Please explain the pros and cons of each method/data source and how can this have impacted your results. For example, administrative data for diabetes can miss cases that are not receiving glucose lowering medications. | We have added two paragraphs to the discussion section addressing these issues. (paragraph 3, the paragraph beginning “The advantage of a self-reported questionnaire” and paragraph 4, the paragraph beginning “Administrative datasets used in our”) |
| The paper mentions high completion rates for self-reported questionnaires. Could you please report the exact response rate in the results section by disease? | The exact response rates have been added to the end of the first paragraph of the results section. (The self-reported questionnaire response rates were: 97% for diabetes, 98% for pre-diabetes, 97% for total diabetes, 96% for hyperlipidemia, 96% for high blood pressure, 96% for mental health disorders, and 97% for asthma.) |
| The paper does not explicitly address potential selection bias. | Unrecognized selection bias may occur because eligible participants who moved overseas may be healthier, and those participants who did not consent to participation or who had died may have an increased burden of illness. However, the demographic characteristics of those who were eligible and those who consented to participation does not suggest any differential bias. We have added a sentence to the “Limitations” section of the discussion to address this. |
| The discussion needs to highlight the limitations, strengths, and future direction. | Thanks for suggestion. All added at the end of discussion section. |
| Reviewer 2 | |
| The introduction lacks a clear explanation of the relationship between antenatal betamethasone and outcome variables. Given that the research explores differences between datasets concerning these variables, the rationale for studying this specific relationship warrants clarification. Is there prior research confirming a link between these variables? Furthermore, evidence of the long-term effects of antenatal betamethasone on outcome variables should be presented if it exists. | An explanation has been added to paragraph 5 of the introduction. |
| The discussion should connect the findings regarding betamethasone and outcome variables with existing literature. | Added as requested to the discussion section. (paragraph 2, the paragraph beginning “The findings of this 50-year follow-up study”). |
| In the results section, I suggest including the p-values alongside the adjusted relative risks and confidence intervals. Instead of presenting the data as 'aRR=1.13, 95% CI 0.56, 2.26', it should be 'aRR=1.13, 95% CI [0.56, 2.26], p-value = xx'. | Thank you for the suggestion, the p-values were added to the Table 3 and the results section. |
| The study frequently cites Shahbaz, M., et al., 'Comparison of outcomes of a randomized trial assessed by study questionnaire and by data linkage: the CONCUR study 2023,' yet the reference lacks a journal name, publication year, DOI, etc. This omission makes the study difficult to locate online. | Details have been added as requested. |
| The manuscript does not address the limitations inherent in self-reported questionnaires and administrative datasets, such as recall bias and the challenge of generalization. | We have added limitations in using self-reported questionnaire in paragraph 3 of the introduction section.  We have already noted the limitation of administrative datasets in paragraph 2 of the introduction section and the way to improve those limitations by combining with self-reported questionnaire in paragraph 4 and 6 of the introduction section. |
| The analysis accounted for two variables (sex and gestational age at the time of the trial). Conditions like diabetes, asthma, and hyperlipidemia are associated with numerous factors, including obesity, family history, and various other clinical and non-clinical factors. Were these factors unavailable in the dataset or not common in various datasets. Whatever the reason, authors should discuss the limitations of the analysis or model in the limitation section. | Because the study was the follow-up of a randomized clinical trial, and characteristics of participants were similar in both groups we have adopted a simple hypothesis testing approach rather than performing mediation analysis, since the exposure may drive intermediate steps and to adjust for them may discount any association between exposure and outcome. The aim of the current study was to determine whether self-reported outcome alone or in combination produced different findings in a trial context, not in relation to a mediation analysis. In the trial context covariates should be apparent at the time of randomization and post-randomization variables should not be include as confounders since they may be on the causal pathway to the outcomes of interest. |
| A dedicated limitations section should be included at the end of the discussion. | Added as requested.  Please see response to reviewer 1 point. |
| The audience may not be as acquainted with the various administrative datasets employed in this study as the authors are. Hence, a concise description of these datasets and the key variables extracted is essential for reader comprehension. | We have added a table describing all datasets. (Table 1)  For extracted key variables please see the appendix 1. |
